# Differential BDNF Responses of Triple Versus Dual Reuptake Inhibition in Neuronal and Astrocytoma Cells as well as in Rat Hippocampus and Prefrontal Cortex

**DOI:** 10.1007/s12031-012-9802-9

**Published:** 2012-05-13

**Authors:** Jos Prickaerts, Jochen De Vry, Janneke Boere, Gunter Kenis, Maria S. Quinton, Sharon Engel, Larry Melnick, Rudy Schreiber

**Affiliations:** 1 Department of Psychiatry and Neuropsychology, School for Mental Health and Neuroscience, Maastricht University, P.O. Box 616, 6200 MD Maastricht, The Netherlands; 2 European Graduate School of Neuroscience (EURON), Maastricht University, Maastricht, The Netherlands; 3 Sepracor Inc., Waterford Drive, Marlborough, MA 01752 USA; 4 Neurobiology Department, Evotec-AG, Hamburg, Germany

**Keywords:** BDNF, Astrocytes, Neurons, Duloxetine, DOV 216,303

## Abstract

Monoamine reuptake inhibitors increase brain-derived neurotrophic factor (BDNF) activity, and this growth factor is regarded as an interesting target for developing new antidepressant drugs. The aims of this study were to evaluate whether monoaminergic reuptake inhibition increases BDNF in vivo and in vitro as predicted by the neurotrophic hypothesis of depression, and whether triple reuptake inhibition has a superior BDNF response compared to dual reuptake inhibition. Twenty-one days of oral treatment (30 mg/kg) with the dual serotonin/noradrenaline reuptake inhibitor duloxetine or the triple serotonin/noradrenaline/dopamine reuptake inhibitor DOV 216,303 restored BDNF protein levels in the rat hippocampus, which were initially decreased due to injection stress. The prefrontal cortex contained increased BDNF levels only after DOV 216,303 treatment. In vitro, neither duloxetine nor DOV 216,303 altered intracellular BDNF levels in murine HT22 neuronal cells. In contrast, BDNF release was more effectively decreased following treatment with DOV 216,303 in these cells. In rat C62B astrocytomas, both antidepressants increased intracellular BDNF levels at their highest nontoxic concentration. C62B astrocytomas did not release BDNF, even after antidepressant treatment. Increased BDNF levels support the neurotrophic hypothesis of depression, but our findings do not clearly evidence that the BDNF response after triple reuptake inhibitors is more effective than after dual reuptake inhibitors. Moreover, the data suggest that the role of BDNF in neurons and astrocytes is complex and likely depends on factors including specificity of cell types in different brain regions, cell–cell interactions, and different mechanisms of action of antidepressants used.

## Introduction

According to the neurotrophic hypothesis of depression, the pathophysiology of depression is caused by reduced neurogenesis, i.e., proliferation and survival of new neurons (Duman et al. 2000; Jacobs et al. 2000). Trophic factors, such as brain-derived neurotrophic factor (BDNF), have been implicated in animal hippocampal neurogenesis, in particular survival (Lee et al. 2002; Sairanen et al. 2005). In the postmortem brain of suicide victims, hippocampal BDNF protein levels are decreased (Karege et al. 2005), and chronic stress in animals decreased BDNF mRNA (Smith et al. 1995) and protein (Xu et al. 2006, 2002) in the hippocampus. In contrast, BDNF mRNA (Calabrese et al. 2010; Nibuya et al. 1995) and protein (Balu et al. 2008; Hodes et al. 2010; Xu et al. 2006, 2002) were increased after long-term administration of several types of monoaminergic antidepressants, such as selective serotonin reuptake inhibitors (SSRIs), serotonin and noradrenaline reuptake inhibitors (SNRIs), and tricyclic antidepressants (TCAs), which primarily act as an SNRI. Likewise, BDNF protein was increased in the hippocampus of depressed patients who were treated with such antidepressants (Chen et al. 2001). Moreover, the same antidepressants increased proliferation and survival of neurons in the hippocampus in animals (Czeh et al. 2007; Khawaja et al. 2004; Madsen et al. 2000; Sairanen et al. 2005; Santarelli et al. 2003; Xu et al. 2006).

It should be noted that not only the hippocampus is implicated in the pathophysiology of depression as BDNF protein is increased in the frontal cortex of rats after treatment with different monoaminergic antidepressants (Balu et al. 2008; Calabrese et al. 2010, 2007; Cooke et al. 2009; Hodes et al. 2010; Mannari et al. 2008). Traditionally, the focus of CNS research is on neurons, but in the last decade, glial cells have received more attention. Studies have reported a decrease in glial cells in the hippocampus (Muller et al. 2001) and prefrontal cortex (Cotter et al. 2002) of patients with depression and in an animal model of chronic stress (Czeh et al. 2007), while chronic antidepressant treatment increased the glial numbers in both structures (Czeh et al. 2007; Malberg et al. 2000; Santarelli et al. 2003).

Evidence is accumulating that BDNF has antidepressant properties. For instance, intrahippocampal injections of BDNF have antidepressant effects in rodent models (learned helplessness) and tests (forced swimming test) of depressive symptoms (Gourley et al. 2008; Shirayama et al. 2002). Along similar lines, it has been demonstrated with an siRNA approach, using an adenovirus, that deletion of BDNF in the dentate gyrus of mouse hippocampus was sufficient to attenuate antidepressant effects of the SSRI citalopram (Adachi et al. 2008). In conditional BDNF knockout mice with a selective BDNF gene deletion in the forebrain, the antidepressant action of the TCA desipramine was attenuated (Monteggia et al. 2007). Thus, BDNF plays a pivotal role in the molecular pathways involved in depression, as well as in the mechanisms underlying antidepressant activity. Therefore, BDNF is regarded as a promising target for studying pathophysiological processes in depression and for developing new antidepressant drugs.

A potential drug development strategy is to further optimize existing drugs known to modulate central BDNF, such as SSRIs and SNRIs. An example is the development of triple serotonin, noradrenaline, and dopamine reuptake inhibitors, such as DOV 216,303 (Skolnick et al. 2003). Demonstration of a superior BDNF response after triple reuptake inhibitor treatment, compared to dual or single reuptake inhibitor treatment, could provide a mechanistic rationale for the hypothesis that triple reuptake inhibitors might be more clinically efficacious.

In the present study, we first investigated the in vivo BDNF response in the hippocampus and prefrontal cortex of rats after chronic treatment with the dual reuptake inhibitor duloxetine and the triple reuptake inhibitor DOV 216,303. In addition, the BDNF response (synthesis and release) of both neurons and astrocytes was studied in vitro by using HT22 hippocampal neuronal cells and C62B astrocytoma cells. The aims of this study were to evaluate: (1) whether monoaminergic inhibition increases BDNF in vivo and in vitro as predicted by the neurotrophic hypothesis of depression, and (2) whether a triple reuptake inhibition approach has a superior BDNF response compared to a dual reuptake inhibitor approach.

## Material and Methods

### Drugs

The dual reuptake inhibitor duloxetine HCl and the triple reuptake inhibitor DOV 216,303-HCl were manufactured by a Sepracor contractor and characterized internally. Both compounds were dissolved in H_2_O.

### In Vivo Experiments

#### Animals

For the in vivo study, 40 healthy male Wistar rats were used (2 months old, 225–250 g; Harlan Laboratories, Dublin, VA, USA). Upon arrival, the animals were acclimatized for 12 days. All rats were housed two animals per cage with food and water ad libitum, controlled ambient temperature, and a 12-h light/dark cycle (lights on, 6 a.m.; lights off, 6 p.m.). The animals were evenly divided into four groups (*n* = 10): control (no treatment), vehicle treatment, duloxetine treatment, or DOV 216,303 treatment. All experimental procedures were in accordance with governmental guidelines and approved by the Board of Registration in Medicine (BRM) Institutional Animal Care and Use Committee (BRM protocol number 03-32).

#### Pharmacological Treatment

On day 13, the treatment period was started at a dose of 30 mg/kg/day duloxetine, 30 mg/kg/day DOV 216,303 or vehicle (H_2_O). These dosages have already been shown to exert antidepressant potential (Breuer et al. 2008; Katoh et al. 1995). The compounds or vehicle were orally administered (by gavage), daily, for 21 consecutive days between 10 a.m. and 2 p.m. Control rats received neither treatment nor handling during the entire treatment period. One animal in the duloxetine group suddenly died on day 4 of the treatment period without any preceding signs of sickness or discomfort (remaining *n* = 9).

#### Sample Collection

Twenty-four hours after the last treatment, all animals were sacrificed by CO_2_; after which, the animals were immediately decapitated, and the prefrontal cortex and hippocampus (bilateral) were isolated and snap frozen in liquid nitrogen. All samples were stored at −80°C.

#### Tissue Homogenization

Frozen brain samples were weighed and transferred into 2-ml screw cap microcentrifuge tubes. For homogenization of the prefrontal cortex and hippocampus, T-PER tissue protein extraction reagent (Pierce Biotechnology, Thermo Scientific, Pittsburgh, PA, USA) was used and supplemented with 1 mM PMSF, 25 U/ml Benzonase nuclease, and complete mini-EDTA-free protease inhibitor cocktail tablets according to manufacturer's instructions. Corresponding volumes of T-PER reagent (500 μl/50 mg tissue) and glass disruption beads (Research Products International Corp., Mt Prospect, IL, USA) (≈0.25 ml beads/50 mg tissue) were added. The tubes were kept on ice for 2 min and homogenized by use of a FastPrep-24 instrument (MP Biomedicals, Solon, OH, USA). Homogenization was followed by centrifugation for 30 min at 4°C and 13,000 g. The supernatant was transferred to new tubes, aliquoted, acid treated (see BDNF immunoassay), and stored at −80°C until used for ELISA.

### In Vitro Experiments

#### Cell Lines

C62B rat astrocytoma (obtained and licensed from Johns Hopkins University, Rockville, MD, USA) and HT22 murine hippocampal cells (obtained and licensed from Salk Institute, La Jolla, CA, USA) were grown on 6-well plates from BD BioCoat Cellware (BD Biosciences, San Jose, CA, USA). Cells were plated at a seeding density of 2.5 × 10^5^ cells/well for C62B (passage 25) and 1 × 10^5^ cells/well for HT22 (passage 10). Both cell lines were cultured in Dulbecco's modified Eagle's medium (American Type Culture Collection (ATCC), Rockville, MD, USA), supplemented with 1 % penicillin/streptomycin (Mediatech, Manassas, VA, USA) and 10 % heat-inactivated fetal bovine serum (ATCC). Cells were maintained at 37°C in a humidified atmosphere of 5 % CO_2_ (C62B cells) or 10 % CO_2_ (HT22 cells).

#### Pharmacological Treatment

Pharmacological treatment was started 16 or 40 h after seeding for 48- and 24-h treatment, respectively. All cells were harvested 64 h after seeding at 80–90 % confluency. For treatment, the culture medium was replaced by fresh medium containing duloxetine (1 or 10 μM) or DOV 216,303 (1, 10, or 50 μM). Of note, higher concentrations up to 100 μM were also tested, but they were found to be toxic (duloxetine was toxic at concentrations of ≥50 μM, while DOV 216,303 was toxic at 100 μM). Toxicity was observed by eye, 100 % free floating cells in the medium compared to 0 % free floating cells at lower drug concentrations. For the negative controls, the culture medium was replaced by fresh medium containing vehicle (H_2_O). All in vitro experiments were performed in triplicate.

#### Cell Harvesting and Protein Isolation

Cells were harvested by use of M-PER mammalian protein extraction reagent (Pierce Biotechnology, Thermo Scientific, Pittsburgh, PA, USA) and supplemented with 1 mM PMSF (Fluka, Sigma-Aldrich, St Louis, MO, USA), 25 U/ml Benzonase nuclease (Novagen, EMD Chemicals, San Diego, CA, USA), and complete mini-EDTA-free protease inhibitor cocktail tablets (Roche, Nutley, NJ, USA) according to manufacturer's instructions.

Culture medium was aspirated, and cells were washed with phosphate buffered saline PBS; after which, 200 μl M-PER reagent was added. After 5 min gently shaking at room temperature, lysates were collected, transferred to microcentrifuge tubes, incubated on ice for 5 min, gently vortexed, and centrifuged at 4°C for 10 min at 13,000 g. For extracellular BDNF measurements, medium was collected and centrifuged for 10 min at 4°C and 13,000 g. The supernatant was transferred to new tubes, and protein concentration was measured by use of the DC protein assay (Bio-Rad, Hercules, CA, USA) according to manufacturer's instructions. Samples were aliquoted, acid treated (see BDNF immunoassay), and stored at −80°C until used for ELISA.

### BDNF Immunoassay

Preceding the ELISA, acid treatment of the samples is recommended for the measurement of BDNF. For this purpose, samples were diluted five times in Dulbecco's PBS, followed by acidification (15 min at room temperature) with 1 N HCl to reach pH < 3.0 and a neutralization step by adding 1 N NaOH to bring the pH to approximately 7.6. After acid treatment, all samples were stored at −80°C until used for ELISA.

Detection of total BDNF protein was performed by use of the BDNF Emax immunoassay system (Promega, Madison, WI, USA) according to supplier's protocol. All samples were assayed in triplicate. To calculate total BDNF protein in cell lysates (picogram BDNF per milligram total protein), ELISA absorbance readouts were corrected for dilution factor and total protein levels. In brain tissue samples, absorbance readouts were corrected for dilution factor and recalculated to nanogram BDNF per gram wet tissue weight as described previously (Balu et al. 2008; Prickaerts et al. 2006). Cell growth medium samples were only corrected for the dilution factor.

### Data Analysis and Statistics

In vivo data were analyzed using one-way ANOVA with compound concentration as a fixed factor and Dunnett's post hoc test. Prefrontal cortex data from one animal in the control group, qualified as an outlier, were discarded from the total data set (remaining *n* = 9). In vitro data were analyzed by two-way ANOVA with compound concentration and duration of treatment as factors. In case of statistical significance, we performed Bonferroni post hoc contrast comparisons to compare treatments with their corresponding control (vehicle) as well as to compare the two treatment durations for each compound concentration. Data are presented as means + SEM, and significance was set at *P* < 0.05. Statistical Package for the Social Sciences (SPSS) 16.0 (SPSS Inc., Chicago, IL, USA) was used for statistical analysis.

## Results

### In Vivo Experiments

**Fig. 1:**
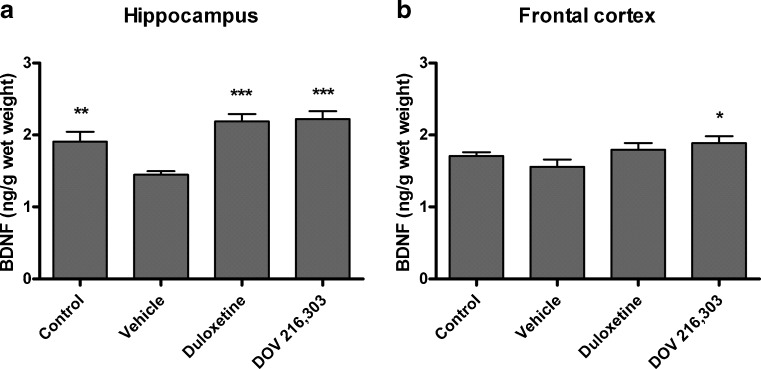
Male Wistar rats were chronically treated for 21 consecutive days with duloxetine, DOV 216,303, or vehicle (H_2_O). Control animals received neither treatment nor handling. The hippocampus (**a**) and prefrontal cortex (**b**) were dissected 24 h after the last treatment, and total BDNF protein levels were measured. Both compounds reversed the reduction in hippocampal BDNF levels due to chronic oral administration. Prefrontal cortex BDNF levels increased after DOV 216,303 treatment only. Data are shown as mean values + SEM. *P < 0.05; **P < 0.01; ***P < 0.001

Chronic treatment of animals with duloxetine or DOV 216,303 had no effect on body weight gain of animals compared to vehicle-treated and untreated control rats (data not shown). Figure 1 summarizes the effects of daily oral vehicle, duloxetine, or DOV 216,303 injections on total BDNF levels in the hippocampus and frontal cortex 24 h after the last administration. There was a significant difference in BDNF levels between groups in the hippocampus (*F*_3,35_ = 11.89; *P* < 0.001). Post hoc analysis revealed that daily administration with vehicle alone (1.45 ± 0.05 ng BDNF/g wet wt) led to a significant reduction in hippocampal BDNF levels compared to the untreated control group (1.91 ± 0.13 ng BDNF/g wet wt; *P* < 0.01; Fig. 1a). Both compounds significantly reversed this oral injection-induced decrease in BDNF levels (duloxetine, 2.19 ± 0.10 ng BDNF/g wet wt; *P* < 0.001; DOV 216,303, 2.22 ± 0.11 ng BDNF/g wet wt; *P* < 0.001; Fig. 1a). In the prefrontal cortex, a tendency was found for differences between groups (*F*_3,35_ = 2.62; 0.05 < *P* < 0.1), which was explained by an increase in BDNF after DOV 216,303 treatment (1.89 ± 0.09 ng BDNF/g wet wt) compared to vehicle (1.56 ± 0.10 ng BDNF/g wet wt; *P* < 0.05; Fig. 1b). No injection effect on BDNF levels was found in the prefrontal cortex.

### In Vitro Experiments

**Fig. 2:**
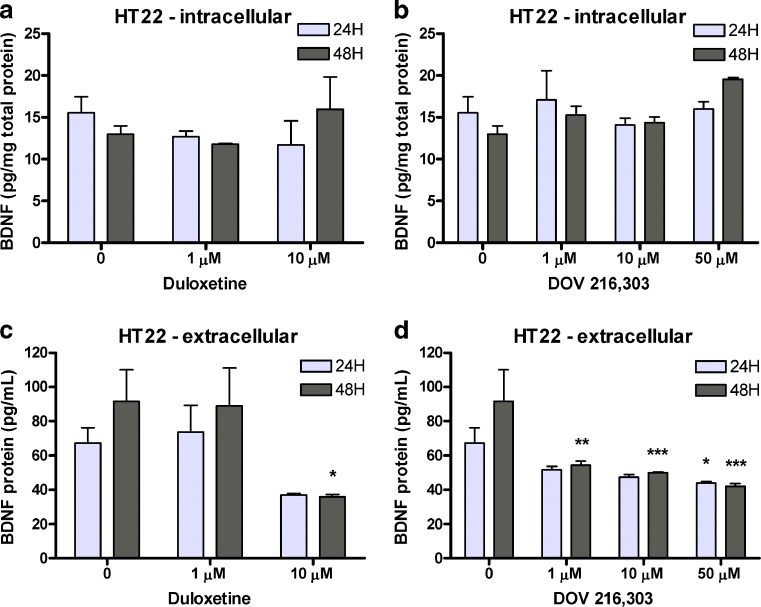
Hippocampal HT22 cells were treated for 24 or 48 h with duloxetine or DOV 216,303. Intracellular BDNF levels were not altered by duloxetine or DOV 216,303 (**a**, **b**). Extracellular BDNF levels were significantly reduced after treatment with duloxetine or DOV 216,303, especially after 48-h treatment (**c**, **d**). Data are presented as mean values + SEM. *P < 0.05; **P < 0.01; ***P < 0.001

Figure 2 summarizes the results for 24- and 48-h treatment of HT22 hippocampal cells with duloxetine or DOV 216,303. Treatment with duloxetine did not alter intracellular BDNF levels in the hippocampal HT22 cells (*F*_2,11_ = 0.67, ns; Fig. 2a), but reduced extracellular BDNF (*F*_2,11_ = 5.41; *P* < 0.05; Fig. 2c). Post hoc analysis showed that 10 μM duloxetine significantly decreased extracellular BDNF levels after 48-h treatment (35.90 ± 1.34 pg BDNF/ml medium) as compared with vehicle treatment (91.52 ± 18.46 pg BDNF/ml medium; *P* < 0.05). Treatment with DOV 216,303 did not alter intracellular BDNF levels (*F*_3,16_ = 2.34, ns; Fig. 2b), but reduced extracellular BDNF levels (*F*_3,16_ = 9.55; *P* < 0.001) at higher DOV 216,303 concentrations after 24 h (vehicle, 67.32 ± 8.93 pg BDNF/ml medium; 10 μM, 47.38 ± 1.43 pg BDNF/ml medium, 0.05 < *P* < 0.1; 50 μM, 44.01 ± 0.76 pg BDNF/ml medium, *P* < 0.05; Fig. 2d) and at lower concentrations after 48 h (vehicle, 91.52 ± 18.46 pg BDNF/ml medium; 1 μM, 54.20 ± 2.54 pg BDNF/ml medium, *P* < 0.01; 10 μM, 49.92 ± 0.46 pg BDNF/ml medium, *P* < 0.001; 50 μM, 42.05 ± 1.60 pg BDNF/ml medium, *P* < 0.001; Fig. 2d).

**Fig. 3:**
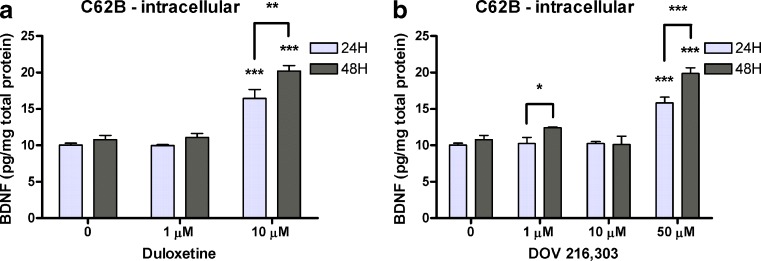
C62B astrocytoma cells were treated for 24 or 48 h with duloxetine or DOV 216,303. Both compounds significantly increased intracellular BDNF levels at their highest nontoxic dose (10 μM for duloxetine and 50 μM for DOV 216,303) (**a**, **b**). At these concentrations, intracellular BDNF levels were significantly higher after 48-h treatment compared to 24-h treatment. No extracellular BDNF was detected in the astrocytes cell cultures. All *bar graphs* represent mean values + SEM. *P < 0.05; **P < 0.01; ***P < 0.001

Figure 3 summarizes the results for treatment of C62B astrocytoma cells with both monoaminergic reuptake inhibitors. Both compounds changed BDNF levels (duloxetine, *F*_2,11_ = 71.98, *P* < 0.001; DOV 216,303, *F*_3,16_ = 56.30, *P* < 0.001). Post hoc analysis showed that both compounds significantly increased intracellular BDNF levels at their highest nontoxic dose (10 μM duloxetine for 24 h, 16.43 ± 1.25 vs. 10.00 ± 0.28 pg BDNF/mg total protein, *P* < 0.001; 10 μM duloxetine for 48 h, 20.19 ± 0.77 vs. 10.79 ± 0.56 pg BDNF/mg total protein, *P* < 0.001; 50 μM DOV 216,303 for 24 h, 15.79 ± 0.80 vs. 10.00 ± 0.28 pg BDNF/mg total protein, *P* < 0.001; 50 μM DOV 216,303 for 48 h, 19.85 ± 0.82 vs. 10.79 ± 0.56 pg BDNF/mg total protein, *P* < 0.001). In addition, 48-h treatment at these concentrations yielded significantly higher intracellular BDNF levels compared to 24-h treatment (10 μM duloxetine 24 vs. 48 h, *P* < 0.01; 50 μM DOV 216,303 24 vs. 48 h, *P* < 0.001; Fig. 3b). No extracellular BDNF was detected in the astrocytoma cell cultures.

## Discussion

The dual reuptake inhibitor duloxetine has antidepressant properties, as found with acute treatment in rodents in the forced swimming test (Katoh et al. 1995). However, to our knowledge, there is only indirect evidence that chronic treatment with duloxetine has antidepressant properties, as it was found to have an anxiolytic effect in mice after chronic treatment (10 mg/kg twice a day for 28 days), but not acute treatment, reflecting clinical experiments with antidepressants in general (Troelsen et al. 2005). The triple reuptake inhibitor DOV 216,303 displayed antidepressant properties in the rat olfactory bulbectomy model when given orally for 14 days at a dose of 20 mg/kg (Breuer et al. 2008), although these data could not be replicated in a similar study (Prins et al. 2011). Our data show a decrease in BDNF levels in the hippocampus of animals that received vehicle by oral gavage as compared to the animals that were untreated controls. Gavage treatment and handling are stressful (Van der Heyden et al. 1997) and likely the cause of decreased BDNF levels, which can be ameliorated with duloxetine and DOV 216,303. The antidepressants increased BDNF in the hippocampus to higher levels than in the vehicle-treated group, but not the nontreated group.

Direct comparisons have shown that the BDNF response seen in the frontal cortex after dual reuptake inhibition was not necessarily observed with single reuptake inhibitors (Calabrese et al. 2007; Cooke et al. 2009; Hodes et al. 2010). This underlines the view that dual substrate inhibition may be more effective than single. Here, an increase in BDNF was observed in the prefrontal cortex only after treatment with DOV 216,303, but not duloxetine, suggesting an even more effective BDNF response of triple versus dual reuptake inhibition. When directly comparing BDNF levels in the frontal cortex to the hippocampus, it can be noted that after chronic treatment with antidepressants like duloxetine, the BDNF protein response in the frontal cortex can be present without any effect in the hippocampus (Balu et al. 2008; Calabrese et al. 2007; Cooke et al. 2009). In the current study, 30 mg/kg duloxetine was apparently high enough to restore BDNF levels in the hippocampus, but was ineffective in the prefrontal cortex. The present lack of an effect of duloxetine on cortex BDNF levels may be due to differences in dissection of the prefrontal and frontal cortex, with different areas of the cortex being present in the two different studies.

Among glial cells, astrocytes are of particular interest as they provide structural, metabolic, and trophic support for neurons (Ransom et al. 2003). Trophic support implies that astrocytes are a source of trophic substances regulating neurogenesis (Song et al. 2002), although they also may contribute to neurogenesis when retaining stem cell-like properties (Horner and Palmer 2003). Support for a role of BDNF produced by astrocytes in the pathophysiology of depression comes from in vivo experiments using conditional BDNF knockout mice with a selective BDNF gene deletion in the forebrain. These studies found that astrocyte-specific BDNF deletion resulted in similar depression-like behavior and attenuation of the antidepressant response to desipramine as with neuron-specific BDNF deletion (Monteggia et al. 2007). In vitro, fluoxetine increased BDNF mRNA expression in primary rat astrocytes within 2 h (Mercier et al. 2004) and in primary mouse astrocytes after 24-h exposure (Allaman et al. 2011). However, 24-h serotonin exposure did not affect BDNF expression in primary mouse astrocytes (Allaman et al. 2011). Yet, monoamine, including serotonin, administration to cultured neonatal astrocytes induced the release of BDNF (Juric et al. 2006). In our study, the undifferentiated C62B rat astrocytoma cells were capable of synthesizing BDNF after treatment with duloxetine or DOV 216,303. The lack of BDNF in the media indicates that these cells did not release BDNF which suggests that the role of BDNF in astrocytes may be dependent on the specific type of cell and its interactions with other cells (e.g., neurons), as well as differences in mechanism of action of the antidepressants used.

In a recent study, the TCA imipramine induced differentiation of cultured rat hippocampal neural stem cells into serotonergic neurons, and this effect was blocked by BDNF siRNA, suggesting that BDNF synthesis and release is necessary (Peng et al. 2008). In the present study, duloxetine and DOV 216,303 were able to decrease extracellular BDNF levels in undifferentiated murine HT22 hippocampal neuronal cells, which are known to express the serotonin transporter, the dopamine, and norepinephrine receptors (Heiser et al. 2002; Schmidt et al. 2001; Shimada et al. 2010). As we expected an increase in BDNF release after antidepressant treatment, this indicates that, as in astrocytes, similar factors are of relevance in determining the net effect of an antidepressant on neuronal BDNF signaling. Of note, there is currently no evidence at hand of a possible physiological function for this decrease in extracellular BDNF levels in our neuronal cells. Nevertheless, the overall effects of DOV 216,303 on the BDNF response in HT22 cells, i.e., its broader range of effective concentrations, might indicate that DOV 216,303 is more effective than duloxetine with regard to the BDNF response in these cells. However, the efficacy is determined by many factors ranging from the presence of monoamine transporters, the occupancy efficiency of the compounds (duloxetine binds better to the serotonin and noradrenaline transporter than DOV 216,303), and the available cell-type specific signal transduction machinery (e.g., DOV 216,303 has an effect on dopamine) to the onset of action (Lengyel et al. 2008). It has to be noted that duloxetine was able to increase intracellular BDNF levels at lower concentrations (10 μM) than DOV 216,303 (50 μM) in astrocytic C62B cells. This would suggest dual reuptake inhibition to be more effective than triple reuptake inhibition in this type of cells. This once more shows that potential differences in efficacy of dual and triple reuptake inhibitors are dependent on the specific biological system being studied.

Our in vivo and in vitro findings as described above indicate that the BDNF response after antidepressant treatment is more complex than predicted by the neurotrophin hypothesis of depression. Some animal and human studies failed to confirm the neurotrophin hypothesis of depression. In human postmortem brain, the effects of antidepressants on proliferation were inconclusive (Boldrini et al. 2009) or not detectable (Lucassen et al. 2010). In animal models of depression, elimination of neurogenesis by focal hippocampal irradiation blocks the antidepressant action of monoaminergic drugs in some (e.g., novelty-suppressed feeding), but not all (forced swimming test) behavioral tests (David et al. 2009). These data suggest neurogenesis-dependent and independent mechanisms of action of antidepressants. This also warrants further research on the exact contribution of BDNF in the pathophysiology of depression and its antidepressant action (Chourbaji et al. 2010).

BDNF is produced as a precursor called proBDNF, which can be proteolytically cleaved to yield mature BDNF (Lee et al. 2001). Whether antidepressants change the production and/or release of proBDNF and mature BDNF is not clear yet, but the few studies that addressed this issue suggest that antidepressants have a limited effect on proBDNF levels (Calabrese et al. 2010, 2007; Mannari et al. 2008). Given the biologically distinct roles of both BDNF forms, with proBDNF linked to apoptotic pathways and long-term depression and mature BDNF linked to enhanced plasticity (Lu et al. 2005), the effects of duloxetine and DOV 216,303 on proteolytic processing of proBDNF are an interesting issue that may warrant future investigation. In this study, however, we could not distinguish between proBDNF and mature BDNF as the antibody from the ELISA we used binds both.

Taken together, our in vivo data show that, in the hippocampus, BDNF levels can be rescued after chronic treatment with duloxetine or DOV 216,303. In the prefrontal cortex, DOV 216,303, but not duloxetine, increases basal BDNF levels. In addition, our in vitro data indicate that intracellular BDNF levels can increase in astrocytes with these monoaminergic reuptake inhibitors. These findings are in accordance with the neurotrophic hypothesis of depression. However, the in vitro data indicate that the effects on BDNF release and, consequently, BDNF signaling are more complex. Together with a lack of a clear in vivo effect in the prefrontal cortex by duloxetine, these data suggest that the eventual net effect on BDNF signaling of a monoaminergic reuptake inhibitor depends on multiple factors, potentially including the mechanism of action of the antidepressant, the particular brain region and its cell–cell interactions. It remains to be elucidated how monoaminergic reuptake inhibitors influence BDNF levels. Increased monoamines activate their respective G protein-coupled receptors, thus activating signaling cascades (e.g., IP3/DAG and cAMP/PKA signaling) that may ultimately result in activation of the transcription factor CREB and BDNF transcription. The routine measurement of in vivo total BDNF or in vitro total intracellular BDNF may not be sufficient to predict the overall neurogenic or antidepressant profile of potential antidepressants. Moreover, evidence suggests that the link between BDNF and its antidepressant efficacy is not as straightforward as was initially anticipated. For instance, studies with BDNF heterozygous mice or with mice having a hippocampal knockdown of BDNF failed to show depressive-like behavior, though such knockdown of BDNF attenuates antidepressant efficacy (Adachi et al. 2008; Monteggia et al. 2007). In addition, it has been shown that BDNF signaling in the ventral tegmental area-nucleus accumbens system exerts prodepressant effects in contrast to the general consensus linking BDNF to antidepressant action (Berton et al. 2006; Eisch et al. 2003). This suggests that the actions of BDNF are highly dependent on the brain region of interest. Some of the data presented in this study hint towards a more effective BDNF response to DOV 216,303, which is “hypothesis generating”, as it suggests that triple inhibitors may possess a more robust neurogenic and, eventually, antidepressant profile. However, sufficient proof to justify such claim is lacking, and this needs to be substantiated with dedicated in vitro and in vivo studies that systematically compare the effects of DOV 216,303 and duloxetine as well as further mono, dual, and triple monoamine reuptake inhibitors. These studies should also measure intra- and extracellular BDNF and distinguish between proBDNF and mature BDNF. Such an approach would result in a composed BDNF response in different standardized settings for monoaminergic or even nonmonoaminergic drugs. This could be used as an indication for their antidepressant potential, which should be verified using a broad battery of behavioral tests.
